# Late effects awareness website for pediatric survivors of acute lymphocytic leukemia

**DOI:** 10.1371/journal.pone.0193141

**Published:** 2018-02-16

**Authors:** Hillary Klonoff-Cohen, Ana Navarro, Elizabeth A. Klonoff

**Affiliations:** 1 University of Illinois Urbana Champaign, Champaign, Illinois, United States of America; 2 University of California San Diego, La Jolla, California, United States of America; 3 University of Central Florida, Orlando, Florida, United States of America; National Cancer Institute, UNITED STATES

## Abstract

**Objectives:**

Every day 43 children are newly diagnosed with cancer. Fortunately, almost 90% of these childhood cancer patients will survive. However, 60–90% of these survivors will experience late effects, health problems that occur months or years after treatment has ended. Late effects could occur as a result of the disease, its treatment, and patient-related factors. The two main objectives of this research are to: 1) Examine the existence of all web-based resources for childhood cancer survivors with acute lymphocytic leukemia which focus on medical and psychological aspects of late effects, and 2) Create an innovative website specifically designed to fill this void.

**Materials and methods:**

A systematic literature review, followed by input from >20 different organizations, resulted in the creation of LEAP^3^ AHEAD (Late Effects Awareness for Patients, Physicians and the Public; Advancing Health and Eliminating All Disparities), a multi-dimensional website centering on late effects.

**Results:**

An extensive review revealed 14 pediatric cancer websites, none of which focused exclusively on late effects. LEAP^3^ AHEAD is the first interactive website for acute lympocytic leukemia childhood cancer survivors and families, as well as physicians, and the public to: a) increase awareness about risks, detection, diagnosis, treatment, and prevention of medical and psychological late effects, b) provide suggestions to successfully reintegrate into schools, careers, and socially, and c) present opportunities including camps, scholarships, and pet therapy programs.

**Conclusion:**

LEAP^3^ AHEAD is the first national website to provide a comprehensive, accessible, affordable, and multi-dimensional resource for pediatricians, internists, nurse practitioners, psychologists, survivors and their families, as well as the public about late effects.

## Introduction

Each year, cancer kills more children between 1 and 20 years of age than asthma, diabetes, cystic fibrosis, and AIDS combined [1]. In 2014, there were 15,780 new cancer cases in children between the ages of 0–19 years [2].

The most common types of cancer in children 0–14 years of age are acute lymphocytic leukemia (accounting for 29% of all childhood cancers), brain and central nervous system (26%), neuroblastoma (6%), Wilms tumor (5%) and non-Hodgkin lymphoma (5%) [3]. Among adolescents age 15–19 years, Hodgkin lymphoma, thyroid carcinoma, brain and central nervous system and testicular germ cell tumors are the most prevalent [4]. Childhood cancer occurs randomly and cuts across all ethnic groups, socioeconomic classes and geographic regions in the U.S.

While treatment advances have increased the survival rate for many childhood cancers, it is still the leading cause of death by disease past infancy among children in the US [5]. Nevertheless, more than 80% of children and adolescents diagnosed with cancer will live at least five years after their diagnosis [6]. Regular, life-long follow-up care is vital because childhood cancer survivors are at risk for late effects. A total of 60–90% of survivors experience late effects [7]—complications, disabilities or other adverse outcomes as a result of the disease, its treatment, or patient-related factors [8] (Table 1). Late effects can occur months or years after treatment, vary widely, and can be physical, cognitive, or psychological in nature. Common late effects include: neuro-cognitive, cardiac, endocrine, and reproductive disorders, obesity and dyslipidemia, bone issues, second malignant neoplasms, and psychosocial problems (e.g., mood, feelings, actions, thinking, learning and memory, social and psychological adjustment) [7].

**Table 1:** Categorization of specific late effects.

Involved system	Specific late effects
Bone marrow	Anemia, polycythemia, leukopenia, marrow hypocellularity, neutropenia, thrombocytopenia, thrombocytosis, other
Skin	Alopecia, atrophy, fibrosis, nail changes, vitiligo, other
Obesity	Obesity (based on BMI), other
Ear	Hearing loss, otitis externa, otitis media, tinnitus, other
Eye	Cataract, dry eye syndrome, glaucoma, retinopathy, uveitis, vitreous hemorrhage, other
Cardiovascular	Cardiac arrhythmia, cardiomyopathy, congestive heart failure, hypertension, hypotension, ventricular dysfunction, other
Lung	Paranasal sinus infection, pneumonitis, pulmonary dysfunction, pulmonary fibrosis, other
Gastrointestinal	Bowel obstruction, colitis, dental abnormalities, chronic enterocolitis, constipation, fecal incontinence, hepatic dysfunction, ileus, malabsorption, mucositis, other
Kidney	Hematuria, hemorrhagic cystitis, incontinence, proteinuria, renal insufficiency, renal tubular disorder, other
Neurologic	Ataxia, cerebrovascular ischemia, cognitive disturbance, dizziness, hydrocephalus, leukoencephalopathy, memory impairment, mood alteration, neuropathy (cranial, motor or sensory), phrenic nerve dysfunction, seizures, speech impairment, tremor, other
Musculoskeletal	Fracture, limb discrepancy, musculoskeletal hypoplasia, osteonecrosis, osteopenia, osteoporosis, scoliosis, other
Thyroid	Hyperthyroidism, hypothyroidism, thyroid nodule, other
Growth	Growth deceleration, growth hormone deficiency, short stature, other
Sexual/puberty	Delayed puberty, gonadotrophin secretion abnormality, gynecomastia, primary gonadal failure, premature menopause, infertility, irregular menses, precocious puberty, other
Metabolic	Adrenal insufficiency, dyslipidemia, glucose intolerance, hypocalcemia, hypercalcemia, other

Childhood cancer survivors are unaware of where to obtain medical care for health issues such as late effects, which may follow cancer treatment [9]. Additionally, pediatricians and other health care providers (i.e., family physicians, internists, nurse practitioners, and physician assistants) may not be knowledgeable about recognizing or caring for late effects [10–11].

There is also a void in the public’s awareness of pediatric cancers and late effects. Currently, there is no resource for physicians, patients or the public that comprehensively address late effects in cancer survivors of different racial/ethnic groups and ages. Given the expanding numbers of pediatric cancer survivors, there is a necessity for developing a mechanism to increase awareness of early detection, treatment, and amelioration of pediatric cancers’ late effects.

Thus, the two main objectives of this descriptive study are to 1) Examine the existence of all web-based resources for childhood cancer survivors with acute lymphocytic leukemia which focus on medical and psychological aspects of late effects, and 2) to create an innovative website specifically designed to educate the general public, pediatricians and other health care providers, and childhood cancer survivors (of all ages) about late effects. The name of this website is **LEAP**^**3**^ **AHEAD: Late Effects Awareness for the Physicians, Patients, specifically, survivors with acute lymphocytic leukemia, and the Public: Advancing Health and Eliminating All Disparities.**

## Materials and methods

### Overview

A systematic literature search of PubMed/Medline and Web of Science core collection databases were performed for studies published until January 2017, using the search terms, childhood cancer, pediatric cancer, pediatric cancer survivors, websites, late effects, long-term effects, childhood cancer resources, and pediatric cancer resources. No relevant articles were identified regarding existing websites on late effects. Hence, Google and Google Scholar were used to locate relevant websites containing information on late effects among childhood cancer survivors. As a final approach, references about existing websites were obtained from our advisory panel, which consisted of community group representatives. They added great richness about how to practically approach, engage, and successfully collaborate with the community in order to develop a meaningful website that appealed to diverse populations. The websites provided in Table 2 are the result of the exhaustive web search and the advisory panel’s expertise in the area.

**Table 2:** Current websites on late effects in childhood cancer survivors.

	Audience	Population	Languages	Disparities Information	Late Effects	Psychological Information	Resources	Appearance
**CureSearch**	Patient, Parent/Family, Community and Friends	Pediatric Cancer Patients and Survivors	English, Spanish	No specific information on disparities in pediatric cancer.	"After Treatment" section consists of various physical complications/side effects, financial and healthcare navigation)	Emotional health as well as risk factors, signs and symptoms, resources for general support and definitions	Several links for other organizational sites, support groups, manual for helping create a follow-up plan	Pleasing format with green color scheme, pictures, print outs available. Four main headings by treatment phase and links by interested party on the right. Drop down menus for age and cancer type when entering a heading.
**Children’s** **Oncology** **Group** **(COG Long-term follow-up guidelines for survivors of childhood, adolescent, and young adult cancers)**	Health care Professionals providing care to survivors of pediatric malignancies	Childhood, adolescent, and young adult cancer survivors	English, some portions translated in French, and Spanish	None	Introduction to problems associated with the heart, dental, endocrine, GI, immune, neurological, pulmonary, and reproductive health after cancer treatment. Includes types of cancer treatment associated with the problem, risk factors for developing problems, precautions and monitoring.	Emotional issues	Several resource links on screening recommendations, and general information on diet and physical activity, education, and payment information for healthcare after completing cancer treatment.	This is not set up as a traditional website. Rather it is resource guide for professionals consisting of a short summary with multiple links on different topics which are downloadable.
**OncoLink**	Lay Individuals, Professionals (nurses primarily)	All cancers, all ages, though predominantly adults	English, Spanish	One fact found on prevalence rates.	Brief information in "Resources for Young Adults" section (basic facts, financial, clinical, etc.)	"Coping" heading with caregiver information, hospice/bereavement, support (support groups, reading room, art gallery, spirituality and inspiration, humor, holiday survival guide)	"Resources for Young Adults"—some overlap with adult information, but few brief facts about childhood cancers, web chat, annual summit information, clinical nurse information, external links (printable)	Format- plain, white background and text with few images. A lot of text, which is cumbersome at times. Set of associated links for each topic area, which is useful.
**Planet Cancer**	Young Adult Cancer Patients and Survivors	Pediatric Cancer Pts and Survivors	English	No direct information but does have an advocacy thread.	Few articles and news feed with some information. Many of the articles are primarily shared personal information or gathered from the public domain and posted.	It is an online cancer support network. Some general information on well-being through personal information, but little specific psychological info.	Many social media resources, both internal and external, including an internal social community. "Cancertainment section". Retreats for survivors, including couple retreats. (printable information). "Welcome to Our World" kit to help with healthcare providers. Financial information and resources for assistance, including scholarship and grant information.	Format- fairly simple with white background and black and red headings and text. Multimedia built into site with online community, videos, blog, and links to Facebook and a Twitter feed.
**Ulman Foundation**	Young Adult Cancer Patients and Survivors	Pediatric Cancer Pts and Survivors	English	Young adults as an underserved population and provide statistics in a report for approx. 2 pages. No other sociodemographic disparity information.	Have a section on education for YA cancer patients and survivors but not many facts about late effects. Do call for advocacy and development of follow-up care for survivors and that is part of their mission. Have various videos and testimonials that have personal information on this topic.	Also has support network resources, and general information about need for support. Little to no specific psychological information. Support information often advocacy related, such as patient decision-making help.	Several external links for support network sites, general cancer information sites, and specific to their alliances (LIVESTRONG, etc.). Videos produced by the foundation and available. Preventative education modules for schools, prevention facts, and prevention links. Activity links such as "Cancer to 5K" and other donor/survivor events. University outreach section, including scholarship information.	Pleasing format with yellow, white, and blue color scheme, pictures of survivors and events, videos, twitter feed and Facebook link, print outs available. Well laid-out in organization and not overwhelming amounts of text.
**Cancer Survivors Project**	Lay Individuals—cancer patients	Long-term cancer survivors (much info geared toward adult survivors of childhood cancer)	English	None available.	Information given via links to books, articles, and websites. Also have brief information by topic provided under "Quick Info/FAQ" link, and much of it is related to late effects (e.g., basic info, health advocacy tips, links to follow-up care plans, etc.).	Very little information. There are references to available information (e.g., 1–2 articles)	"Reading List" link with information for books, articles, and websites for helpful information. Also includes a "How To" section on researching information. Health care provider list by state of clinics that treat long-term survivors. Listserv, survivor stories,	Very plain in layout. Able to navigate easily.
**American Childhood Cancer Organization (Candlelighters)**	Communities, Families, Cancer Pt's and survivors, Advocates	Pediatric Cancer Pts and Survivors	English	None found.	Have a section in the "Information" table on Late Effects with several links to topics within the site and also external links to organizations and articles.	Have a "Support" heading, which includes financial information and resources, "Remembered Forever" section, bereavement (just links to outside groups) and approximately one page of information on the topic psychological and emotional. This has information for local affiliates who may have face-to-face support groups, online support group links, and brief information on potential referral sources if counseling is wanted.	Very cool iCancer app for keeping your medical care organized. Local affiliates who provide services and help to families. A helpful book for parents and teachers about education and childhood cancer pts. "Advocacy" heading with several links to advocacy organizations, information on how to write your officials, current and past information about legislation, and research-based info. Also have "Awareness" heading with resources for events, organizations for promoting awareness, both internal and external to the group. Information and links for kids and teens, as well as information and links for siblings. Books, newsletters, websites for more information.	Pleasing format with very clean and concise look. Another yellow, blue and white background website. Headings are across the top and when highlighted have drop down menu trees for further information headings. Cool peel-back feature at the top, right hand corner for sponsoring or donating for an event. Homepage has an inspiring video for the annual awareness/celebration of tree-lighting. Also have icons to link to their Facebook, Twitter, and YouTube accounts.
**Fertile Hope**	Cancer Patients and Health Professionals	Cancer Patients and Survivors	English	None found.	Fertility being a specific late effect. They do have a section on fertility information specifically for pediatric cancer pts and survivors (2–3 pages, by gender).	They have a link to the LIVESTRONG site where there are resources for support and some information, but nothing specific about psychological effects. It is embedded in some of the information in a general way.	Have a guide for fertility information and resources, which includes resources for finding healthcare, CAM therapies, fertility preservation, etc. by zip code and you can read about the providers and services. Also, have cancer centers of excellence for fertility. Have a risk and options calculator by cancer type and treatment, which shows the risk of developing infertility. One of their major purposes is to help people find financial resources to help with fertility preservation, such as adoption services, sperm and egg preservation, etc. Also, have resources for health professionals, including information, fast facts print outs, options to present about the topic, and research from the field. There is also a "kit" available to help facilitate cryogenic freezing and a link to the website for the kit. They also have the standard resources page with links to organizations and readings about fertility and cancer.	Basic format, easy to follow with major headings listed on the left and tabs on the right for resources guide, calculators, and "kit". Easily navigated and tools are easy to use as well. The resource guide is laid out very well and lots of searching terms available.
**National Coaltition for Cancer Survivors**	Cancer survivors, families (very driven toward pt education and advocacy)	All cancer types, all ages	English, Spanish, and toolbox is available in Spanish audio, transcripts in Chinese	None found.	Not specific information for pediatric late effects, but some general information about late effects of blood cancers.	Have a good amount of information on the emotional aspects of cancer, including case examples and information about talking to your healthcare provider about these topics. Part of the audio and pdf toolkit in "Special Topics". Have information about intimacy as well in the larger section.	Award-winning audio cancer survivor toolkit (available direct from site, downloadable on iTunes, and CD purchase), with a facilitators manual available for healthcare professionals to download for free. A resources guide for a condensed version in a brochure form of the toolkit. Lots of great tips and tools to advocate for your healthcare as a survivor. A few survivor testimonies available too. Searchable resource guide with drop-down menus for location, cancer type, issue, etc. Also an advocacy toolkit. Cancerversary.org link and info to join a social network and create your own page to commemorate. Survivor profiles and stories.	Nicely laid out and easy to navigate. Have cross-links to topics so that you can get there multiple ways by related topics, or through the homepage headings. Accessible pdf's and audio files, as well as a few video files. Not very many graphics or intriguing visual aspects, but straight forward and clean.
**Leukemia and Lymphoma Society**	Cancer patients and survivors, families, and scientists and professionals	Leukemia and Lymphoma cancer patients and survivors	English and Spanish	None found.	Have some information about survivorship and late effects in general with links out to guidelines for care and more information. Not many specifics, and mostly links out.	Have a section on "How to help your child cope", (1pg) also how to talk to your child about leukemia/lymphoma, and sibling and parent coping (1pg'ers).	Have links to several resource guides about survivorship and guidelines for treatment of late effects. Advocacy resources such as action alerts and email-writing tools to your congress person, and a toolbox for advocates. Information for financial assistance, finding support, getting information, a discussion board, have an information center call-in, a new dx peer support program, and information that is printable (like fact sheets, etc.).	Has a lot of information and feels a bit overwhelming and crowded in format. Not too difficult to navigate, but a big contrast to something like the Ulman Foundation which provides a lot of info but has less going on so it "appears" easier to navigate.
**Cancer Information Counseling Line**	Lay Persons- Cancer pts and families	All cancers, all ages	English	None found.	None specifically.	This is a support line for all people with cancer. Open to anyone to call in and get support for any stage of treatment and/or survivorship. Open from 8–4:30pm MST.	It is a resource, but also will help those who call in attain resources that they may need for treatment.	It is part of a larger hospital site. Very brief page of information, mostly text, with bullet points.
**KidsHealth http://kidshealth.org/**	Parent/Family, cancer survivors	childhood cancer survivors	English	None available.	Discusses the causes of late effects and provides detailed examples of common late effects experienced by childhood cancer survivors.	Have a section titled, "Dealing with uncertainty", but other than this section does not go into much detail on the psychological effects.	At the bottom of the page, there is a box where viewers can go to for more information on the topic. It is subdivided into three different tabs, "For parents, for kids, and for teens", providing relevant information for that age group.	Very plain layout, but it is easy to navigate. There is a lot of information provided in the three pages, yet very few pictures.
**National Children's Cancer Society https://thenccs.org/**	Childhood cancer survivors, parent/family	childhood cancer survivors	English	None found	There is a tab under the "Survivorship" section titled, "Late Effects After Treatment Tool". This is a resource where cancer survivors can either input information on their own personal cancer diagnosis/treatment and receive information on late effects specific to them, or choose to not to put in specific information on their treatment and instead have an assessment on their medical concerns. The LEATT takes less than 10 minutes to complete and describes potential late effects specific to the patient, symptoms to watch for, and recommendations/prevention tips to look out for.	In the Survivorship section, there is a tab that provides information for both patients and parents. One of the categories viewers can click on in both is titled, "Emotional Information". It discusses the types of emotions survivors may face, how to deal with stress and anxiety, post-traumatic stress disorder, how to deal with depression, and information about attending a support group.	There is an entire section titled, "Education and Resources “that directs patients and parents to publications, conferences, webinars, helpful links, the late effects after treatment tool, links for long term follow up clinics, special education information, educational articles and cancer facts.	Plain layout with a white background and blue headings. It is very well organized and easy to follow with the main categories at the top. Instead of having to scroll through an entire section for the information you are looking for, the category is subdivided at the top with the different headings discussed.
**Cancer.net**	Childhood cancer survivors but geared towards surviving cancer in general. For example, if you survive breast cancer and liver cancer the same links are applicable to both of those cancers if you are an adult.	All cancer types, all ages	English	None found	There are two sections under the "For Children" page on the website titled “Late Effects of Childhood Cancer" and "Managing Late Effects of Childhood Cancer." The Late Effects section gives a list of the causes of late effects and a brief description of each type of late effect. The late effects included range from emotional and learning troubles to physical setbacks such as dental and digestive issues. The Managing Late Effects section goes through what to do before, during and after treatment as well as coping and lowering late effects. For example, before treatment includes a list of questions the patients should ask their doctors while the after section includes checkups and screening tests at follow-up clinics.	In the Managing Late Effects section there is a brief description of learning and memory problems as well as emotional troubles that patients may deal with. The information in this section includes that patients may have anxiety, depression, and fear of recurrence. It also includes that patients with memory and learning problems may need referrals school programs or state and county social services. In the "Coping with Cancer" tab there are managing emotions, talking with friends and family, and finding support and information that includes information on how to find counseling and support groups. However, this section is not specifically for childhood cancer survivors and applied to all cancer survivors.	Under the Coping with Cancer tab there is a section titled "Finding Support and Information" that has a subheading "Cancer Specific Resources" that allows users to enter in the cancer they are looking for and the results bring up Cancer Groups and Foundations. For example, upon entering Childhood Cancer into this area, the groups and foundations presented included 21 different childhood cancer foundations with links to their websites and a phone number to contact. Some examples of the foundations are Locks of Love and the Make-a-Wish Foundation.	The appearance of this website is not very exciting. It is plain white with yellow headings and blue text. I found this website very difficult to navigate. All the information pertaining to one type of cancer could not be found in the same area. For example, for childhood cancer there one link but it does not include the resources for Late Effects or Emotional/Psychological Help. That information is in a completely different part of the website. However, this part does include statistics (how many children get and die from childhood cancer), overviews, risk factors, prevention, clinical research, symptoms and signs of childhood cancer.
**Cancer.orghttp://www.cancer.org/treatment/childrenandcancer/whenyourchildhascancer/children-diagnosed-with-cancer-late-effects-of-cancer-treatment**	Parents and loved ones of a childhood cancer survivor	The entire website is for all cancer types and all ages but this specific page is for childhood cancer.	English	The only information found was the difference in treatment effects on sexual development and fertility based on gender.	Late Effects is an entire section for the Cancer.org website. This particular page is specific to childhood cancer and discusses who is at risk for late effects, what causes late effects, treatment effects on parts of the body, treatment effects on growth and development, treatment effects on sexual development and fertility, emotional issues, and follow-up guidelines.	The psychological information they include on this page is under two different sections. The first is Brain, which has information about learning disabilities and cognitive impairments. Under the Emotional Issues heading, the information included regards dealing with physical changes, worries about the cancer returning, feelings of resentment, concerns about being treated differently, and concerns about dating, family life, etc. This section does not offer solutions to these issues, it only states that childhood cancer survivors may experience them.	The resources this website offers are pages with more information which include: Fertility and Women with Cancer, Fertility and Men with Cancer, Second Cancers caused by cancer treatment, Cancer information on the internet, Children Diagnosed with Cancer (include subcategories of Dealing with Diagnosis, Financial Issues, Understanding the Health Care System, and Returning to School), and When you're child's treatment ends: A guide for families. In addition to these pages, the website also offers links to other national organizations and specific websites for children and teens to reach out to others.	The format of this page is very formal and unpleasant. The font is black and there is not very much color added to the different headings. There is one picture at the top but it does not seem to be specific to this page and you cannot see everyone's face in the picture. I think the sections on this page should be separated to completely different pages because there is too much information crowding in one area.

The advisory panel reviewed and critiqued the description and evaluation of the websites.

They determined whether the websites were: 1) specific for late effects among childhood cancer survivors, 2) user friendly, 3) attractive, 4) easy to comprehend for the specific sample, and 5) sources for relevant resources. The advisory panel were vigilant about creating a website that appealed to all ages, educational abilities, with the option to translate into several languages. The general public was included to increase awareness about late effects among the entire population.

### Development of the LEAP^3^ AHEAD website

The LEAP^3^ AHEAD website was developed after comprehensively reviewing the literature and receiving input from a coalition consisting of both existing and new organizations in San Diego. The existing group included pediatric oncologists at Rady’s Children’s Hospital, and members from several established organizations, including American Cancer Society, MANA (short for “hermana,” the only national Latino organization that focuses on pediatric oncology), Union Pan Asian Community, and San Diego Black Nurses Association. The new organizations consisted of Community Health Improvement Partners, Starlight Children’s Foundation, and the Family Resource Collaborative (containing 20 organizations in San Diego, including Make A Wish, Leukemia and Lymphoma Society, ROCK cancer, and the County Health Department). Additionally, the coalition contained three childhood cancer survivors.

The members of the advisory group were chosen because of their interest and dedication to pediatric cancer, their willingness to participate, their availability to attend meetings, and their location (residing in California). All meetings were held at the Rebecca and John Moores Comprehensive Cancer Center, UCSD from January 2011 to May 2013. The participating organizations provided input and suggestions related to the website contents and layout, cultural and linguistic suitability, educational and outreach materials, translational and cultural adaptation, dissemination of the information, and website evaluation over the course of one year. After multiple revisions, the final website was completed, pilot-tested by cancer survivors, family members, and people in the community, and then refined.

#### Set up of website

The contents of the website were developed for five different childhood cancer survivor age groups including: youth (0–10 years) and their parents, tweens (11–17 years), young adults (18–25 years), adults (> 25 years), as well as for physicians and the public based on feedback from the advisory committee, people in the community and parents and childhood cancer survivors.

Symptoms and diagnosis, treatment, and prevention of late effects are featured on the website under the physical and emotional health (medical) tab for each age group. By hovering the mouse over a particular part of the body where the individual is experiencing pain or discomfort, the web user can easily identify their symptoms and potential diagnosis. The participant is able to click on dental, cardiac, endocrine, gastrointestinal, musculoskeletal, neurological, pulmonary, reproductive, and sensory systems. For example, if the survivor suffers from a headache, they would move the mouse to the head or neurological part of the diagram and the pop-up box would lead them from symptoms to a potential diagnosis, treatment, and preventive strategies. At present, the medical information is directed towards acute lymphocytic leukemia, since it is the most common type of pediatric cancer. In the future, other types of pediatric cancers will be included in the website.

Reintegrating back into life after completing cancer treatment can be challenging for childhood cancer survivors. For child, tween, and young adult survivors there are obstacles when interacting with friends, coworkers, and families as well as returning to/re-entering school or university or the professional environment (e.g., work setting). LEAP^3^ AHEAD provides potential suggestions for cultivating relationships and achieving success at work and school. Information on other opportunities including pediatric cancer survivor camps, scholarships, alternative care (yoga) and companion animals are also offered on the website. Finally, the website contains music and contributed art and poetry created by pediatric cancer survivors.

#### Appearance

Efforts were made to create an interactive website with an attractive logo, tag line, bright colors, interactive images, which were approved by the participating organizations. The website has two links entitled “Educate Yourself,” and “Educate Your Community.” The latter provides a ready-to-use educational outreach packet for community leaders to post, hand out, place in a newsletter, or use in a talk.

All website and written materials were checked for content, literacy demand (e.g., reading grade level, vocabulary, learning aids, graphics, and layout) learning stimulation and motivation, and cultural competence. The messages were consistent, recognizable, and used language that enhanced health literacy. All materials were translated into Spanish. In the future, this website will be translated into additional languages, such as Chinese, Tagalog, Japanese, and Korean.

#### Target population

The initial target population consisted of San Diego and Imperial County residents, which has a diverse background, characterized by various cultural beliefs, socioeconomic conditions, and languages. The website was targeted towards cancer patients/survivors and their families, residents who are interested in this information, those at high risk for developing childhood cancer, as well as all individuals who would benefit from an enhanced awareness of childhood cancer late effects. Particular attention was devoted to underserved populations, including those who lack adequate health insurance.

The second audience consisted of the professional community, who also required increased awareness and education. They include groups involved in any aspect of childhood cancer prevention, detection, or treatment, including health practitioners, nurse practitioners and physician assistants, community leaders, and faith-based leaders, as well as professional societies and volunteer organizations such as St. Baldrick’s Foundation, LiveStrong, and the American Cancer Society. These groups require increased cultural competency to provide patients with the best standard of care.

#### Dissemination

In order to effectively reach low income and ethnically diverse segments, media communication venues will be utilized as well such as newspapers, magazines, flyers, radio, and TV. This includes Twitter, Facebook, Instagram, and Snapchat in order to reach younger generations.

## Results

### Extensive literature review of existing web-based resources

There are several well-established websites and programs for childhood cancer survivorship, but none devoted exclusively to late effects, with resources available to survivors, families, health care providers and the public. Currently, there are fifteen websites in existence (Table 2) that contain some information on late effects; albeit, this is not the main focus of any of the databases. Rather, they are based on childhood cancer and directed towards diverse audiences including cancer patients and survivors, families and friends, scientists, researchers, professionals, and advocates. Table 2 contains summary information regarding the source, targeted audience(s) (e.g., patients, physicians), population (s) of interest (e.g., cancer patients vs. survivors), translation into additional languages, disparities, medical and psychological late effects, available resources, and overall appearance of the fifteen websites. All websites concentrate on pediatric cancers survivors, primarily in their twenties, apart from the Ulman Foundation, which centers on adolescents and young adults with cancer (AYA) and Children’s Oncology Group which encompasses pediatric, adolescent, and young adult cancer survivors. CureSearch targets the greatest audience, including patient, family, friends, but not the general public.

Only one website, Ulman Foundation, broaches disparity. They describe young adults as an underserved population and provide two pages of statistics. No other website tackles disparity related information. CureSearch contains the greatest detailed information for cancer survivors on the most common late effects, specifically medical complications categorized by organ system (but not by specific type of childhood cancer), finances, and healthcare navigation (e.g., finding a healthcare team, paying for healthcare). In contrast, KidsHealth and National Children’s Cancer Society explain late effects specific to the patient’s own cancer diagnosis and treatment. Children’s Oncology Group is superior for providing extensive information for health care professionals. Cancer.org discusses who is at risk for and what causes late effects, treatment effects on growth and development, fertility, and emotional issues. Fertile Hope deals exclusively with fertility related late effects. The National Coalition for Cancer Survivors contains no specific information for pediatric late effects, other than on blood cancer late effects. Additionally, the Leukemia and Lymphoma Society, Cancer Information Counseling Line, Kids Health have no information on late effects. Limited psychological and emotional late effect information is provided in the following websites: CureSearch, Ulman Foundation, American Childhood Cancer Organization, Cancer.net, and Cancer.org. Additionally, CureSearch, OncoLink, American Childhood Cancer Organization (Candlelighters), Cancer Information Counseling Line, and Cancer.net offer support group resources. However, these support groups are not specifically for pediatric cancer survivors, apart from the National Children’s Cancer Society.

### Program evaluation

Individuals visiting the LEAP^3^ AHEAD website will have the opportunity to provide feedback using a short, voluntary, pop-up survey where information will be collected about relevant demographics, how the individual discovered this website, and if the information was helpful.

The primary outcomes of interest to assess the impact of the program include (but are not limited to) the following: i) Who is using the website (e.g., public, cancer patients, family members, professionals), ii) Their demographics (e.g., age, race, ethnic background, gender, formal education level, occupation, primary spoken language, use of English or translated version of the website, place of residence, and health status); iii) Perceptions about the ease of use and content of the website; iv) If they will return to the website and reason(s), particularly for recognition of late effects or treatment protocols; and v) If they will refer somebody to the web site. Each of these questions will be examined by overall and racial/ethnic sub group where appropriate.

If the person is a patient/parent, they will be queried about whether the website information: i) assisted them in accessing medical or psychological treatments, ii) resulted in them practicing any of the preventive options; and iii) increased their awareness about opportunities (e.g., camps, dog companionship). In addition, website usage statistics will also be examined. Of particular interest is an evaluation of which content is viewed most frequently and where appropriate, tagged, re-posted or emailed to Facebook or to other friends.

## Discussion

Currently, there are 15 websites that contain some information on late effects. For example, CureSearch has a section, “After Treatment” which discusses various physical complications and side effects of cancer treatment. Children’s Oncology Group has a description of only the medical aspects of late effects associated with heart, dental, endocrine, GI, immune, neurological, pulmonary and reproductive systems. The National Children’s Cancer Society has a resource where cancer survivors can input information on their own personal cancer diagnosis and receive information on possible late effects specific to them. Late effects are not the sole focus of any of the websites.

Coping with late effects throughout survivorship requires young survivors to continuously appraise their cancer’s threat and its potential for change at various times throughout the remainder of their life [12], including negative effects on reproduction, re-interacting with friends or family members and formulating meaningful relationships, adjusting to changes in self-esteem and physical capabilities, and fearing a recurrence or second diagnosis of cancer [12]. LEAP^3^ AHEAD addresses all these challenges within the context of the life course. For example, during critical periods of youth, tween, young adulthood, and adulthood, disruptions with peers and school may occur. A 17-year old survivors’ sense of well-being may be founded on an ability to establish independence in life or perform well in school, whereas achieving expected life goals such getting married or having children may have greater salience for a 30-year old survivor’s well-being [12].

Hence, this website is the first to provide age specific, audience specific, and language specific (bilingual) information to: a) assist childhood cancer survivors, families and their caregivers, and the public about the timely recognition of late effects and medical, emotional, neurocognitive, and psychological signs/symptoms and prevention strategies throughout the lifespan, and b) provide suggestions about how to reintegrate childhood cancer survivors socially with family and friends as well through education and employment prospects. At this point, the evaluation for reintegration has not occurred so it is unclear whether these suggestions will have an impact on childhood cancer survivors.

Nevertheless, it is problematic for childhood cancer survivors to reintegrate socially when they return back to school. Long absences and missed work could result in students feeling unprepared. Moreover, children may experience physical changes (e.g., amputation, hair loss) [13], feel depressed, anxious, rejected by their pears, and isolated. Teachers may lack knowledge about cancer, have unrealistic expectations, and may be unprepared to handle classmates’ reactions [13]. Finally, parents may be fearful about sending their children back to school because of concerns about infection, medical emergencies [14], teasing by other children, and over-protectiveness.

The following examples from the website consist of social interactions for Friends-children until age 10, and School-children until age 10.

Regular follow-up care by health professionals who are trained to recognize and treat medical and psychological late effects is also extremely important for the long-term health of childhood cancer survivors. Hence, pediatricians, family physicians, psychosocial providers and internists may utilize this web-based resource within their practices and refer patients to LEAP^3^ AHEAD.

Furthermore, childhood cancer survivors may gravitate towards risk-taking behaviors [15]. LEAP^3^ AHEAD has a prevention section which educates survivors about indulging in healthy lifestyles (i.e., healthy diet, exercise, regular medical and dental checkups), while avoiding tobacco, alcohol and illicit drug use, and exposure to sunlight.

## Conclusion

By 2020, an estimated 500,000 survivors of childhood cancer are expected to be living in the U.S [16–17]. Decisions made by doctors and parents could impact these children throughout their lifespan. Currently, in order for survivors to partake in childhood cancer survivorship programs they must have survived at least 2 years to 5 years since completion of all cancer therapy. If this criteria is met, then participants receive only yearly evaluations by a team of professionals specializing in cancer including, but not limited to, a psychologist, physician, nurse practitioner, and dietician. Because of the paucity of survivorship clinics nationally, strict criteria excluding entry directly after completion of treatment, and limited follow-up (annually), it is advisable to offer other complementary modalities to childhood cancer survivors. Furthermore, lower risk childhood cancer survivors are often transitioned back to their primary care physicians to continue lifelong follow-up. Often, adult -focused primary care providers lack survivorship expertise due to the paucity of survivorship-related content in medical school curricula and residency training.

To date, LEAP^3^ AHEAD is the first national website to provide a comprehensive, accessible, affordable, and multi-dimensional approach about late effects for pediatricians, internists, nurse practitioners, psychologists, childhood cancer survivors and their families, as well as the public. This website could be of value to childhood cancer survivors and providers. LEAP^3^ AHEAD evaluation is in its beginning; thus, information on its effectiveness and impact will be provided in future reports.

## Supporting information

S1 Graphic Friends-children, school-children (until age 10). Important considerations and helpful solutions regarding friends and school, for children until age 10.
(DOCX)
