# Supplementary figures and images for: Late effects awareness website for pediatric survivors of acute lymphocytic leukemia
Source: PLoS One. 2018 Feb 16;13(2):e0193141. doi: 10.1371/journal.pone.0193141 (PMC5815604; doi:10.1371/journal.pone.0193141)

**Friends-Children, School-Children (until age 10).**


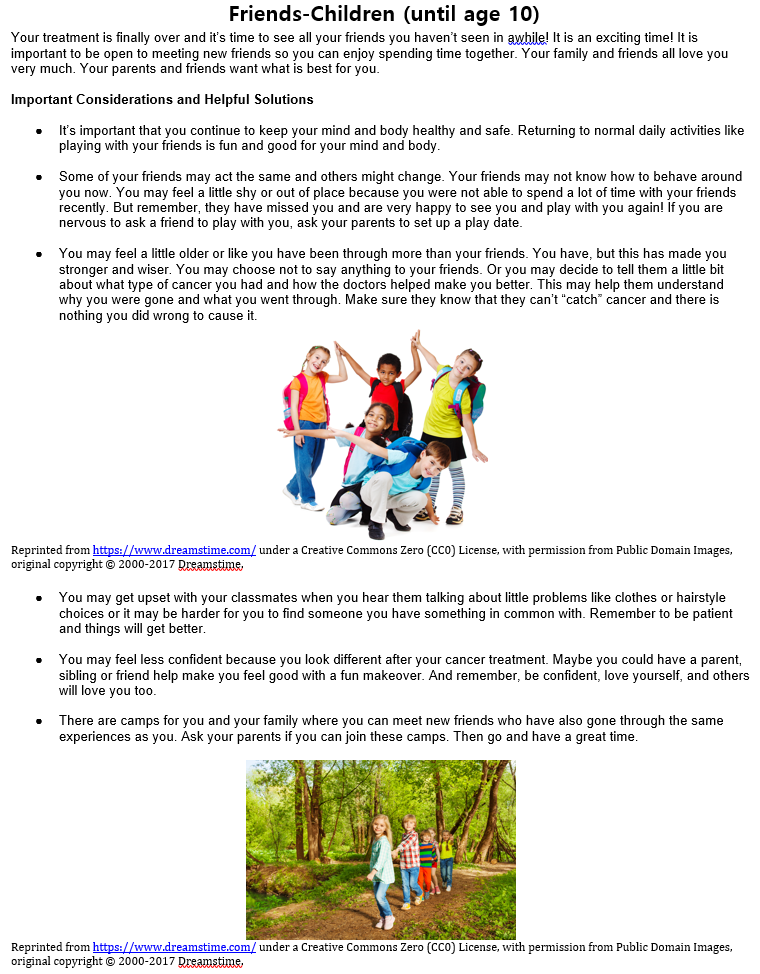


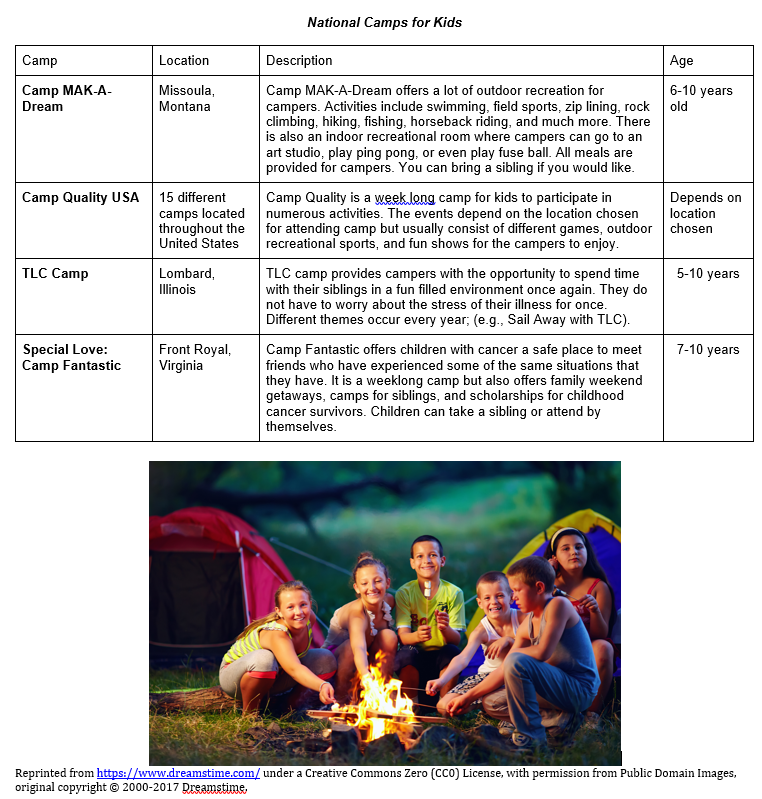


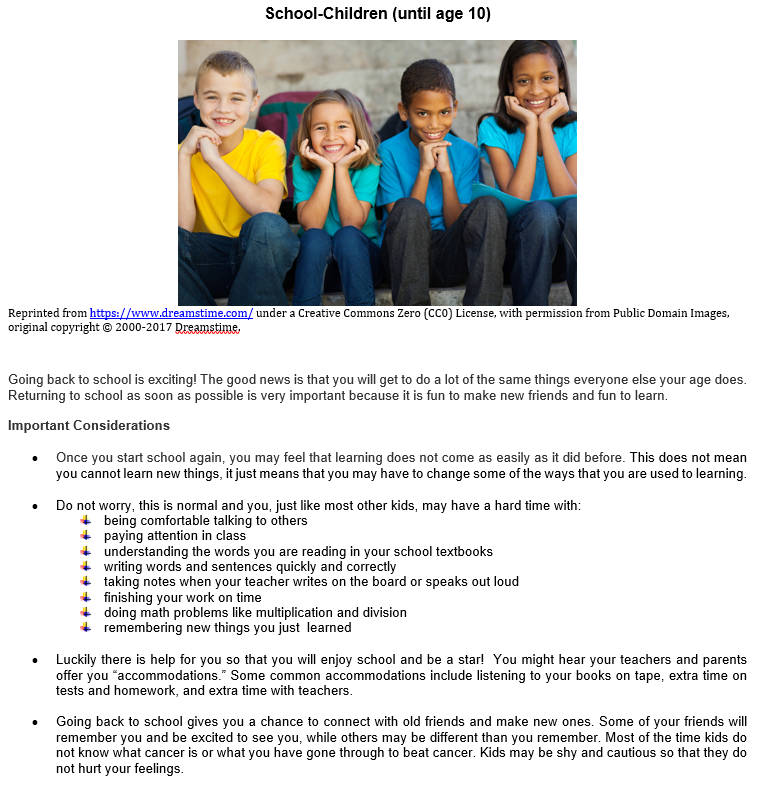


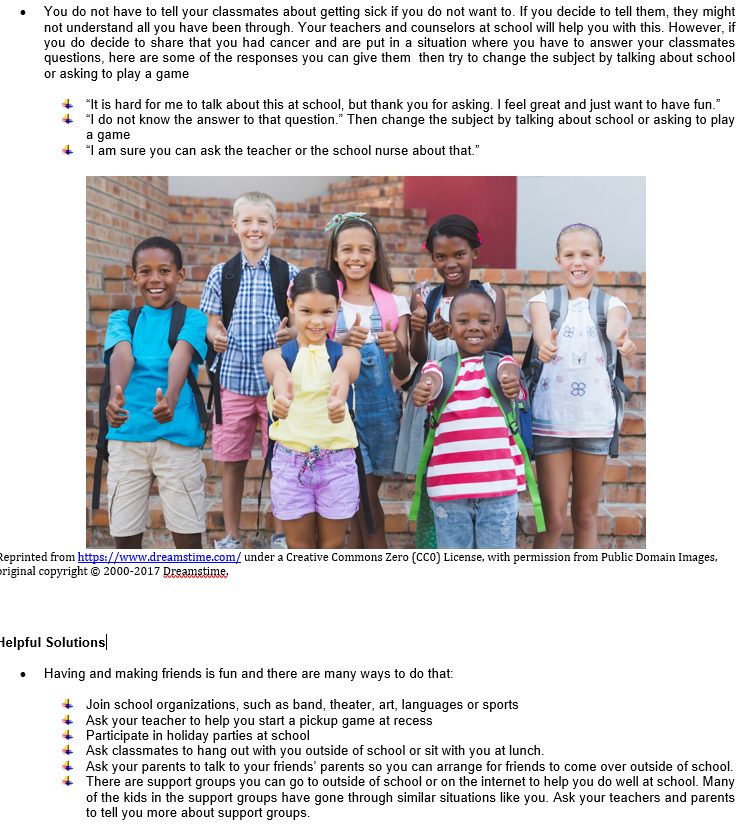


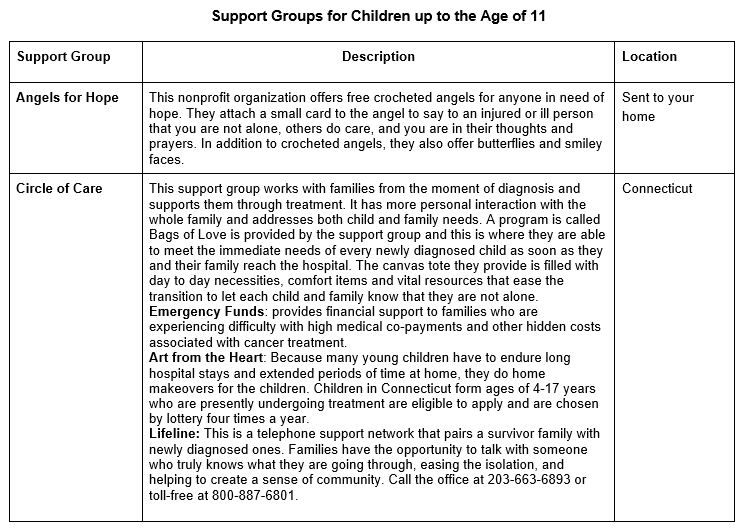

Supplement: S1 Graphic — Important considerations and helpful solutions regarding friends and school, for children until age 10. (DOCX) [file pone.0193141.s001.docx]
